# Structural Basis of Mucopolysaccharidosis Type II and Construction of a Database of Mutant Iduronate 2-Sulfatases

**DOI:** 10.1371/journal.pone.0163964

**Published:** 2016-10-03

**Authors:** Seiji Saito, Kazuki Ohno, Torayuki Okuyama, Hitoshi Sakuraba

**Affiliations:** 1 Department of Medical Management and Informatics, Hokkaido Information University, 59–2 Nishinopporo, Ebetsu, Hokkaido 069–8585, Japan; 2 Catalyst Inc., 1-5-6 Kudan-minami, Chiyoda-ku, Tokyo 102–0074, Japan; 3 Education Academy of Computational Life Sciences, Tokyo Institute of Technology, 2-12-1 Ookayama Meguro-ku, Tokyo 152–8552, Japan; 4 Department of Clinical Laboratory Medicine, National Center for Child Health and Development, 2-10-1 Okura, Setagaya-ku, Tokyo 157–8535, Japan; 5 Department of Clinical Genetics, Meiji Pharmaceutical University, 2-522-1 Noshio, Kiyose, Tokyo 204–8588, Japan; University of Naples Federico II, ITALY

## Abstract

Mucopolysaccharidosis type II (MPS II, Hunter syndrome) is an X-linked genetic disorder caused by a deficiency of iduronate 2-sulfatase (IDS), and missense mutations comprising about 30% of the mutations responsible for MPS II result in heterogeneous phenotypes ranging from the severe to the attenuated form. To elucidate the basis of MPS II from the structural viewpoint, we built structural models of the wild type and mutant IDS proteins resulting from 131 missense mutations (phenotypes: 67 severe and 64 attenuated), and analyzed the influence of each amino acid substitution on the IDS structure by calculating the accessible surface area, the number of atoms affected and the root-mean-square distance. The results revealed that the amino acid substitutions causing MPS II were widely spread over the enzyme molecule and that the structural changes of the enzyme protein were generally larger in the severe group than in the attenuated one. Coloring of the atoms influenced by different amino acid substitutions at the same residue showed that the structural changes influenced the disease progression. Based on these data, we constructed a database of IDS mutations as to the structures of mutant IDS proteins.

## Introduction

Iduronate 2-sulfatase (IDS, EC 3.1.6.13) is a lysosomal enzyme that catalyses the hydrolysis of sulfated esters at the non-reducing-terminal iduronic acids in the glycosaminoglycans (GAGs) heparan sulfate and dermatan sulfate. A deficiency of IDS activity results in systemic accumulation of GAGs, leading to a rare metabolic disease, mucopolysaccharidosis type II (MPS II, OMIM 309900), which is also known as Hunter syndrome [1]. Patients with MPS II typically exhibit systemic manifestations including a short stature, a specific facial appearance, dysostosis multiplex, a thick skin, inguinal hernia, hepatosplenomegaly, hearing difficulty, an ophthalmic problem, a respiratory defect, heart disease, and occasional neurologic involvement. However, this disease exhibits a wide range of clinical phenotypes from the “severe form” with progressive clinical deterioration to the “attenuated form” without mental retardation.

Enzyme replacement therapy (ERT) involving recombinant human IDS is approved in many countries for treatment of MPS II patients. As recent studies demonstrated that ERT improved the manifestations of MPS II, especially when it was started at an early age [2–5], an early diagnosis and clinical phenotype determination are becoming more and more important for predicting the prognoses of patients and a proper therapeutic plan.

The IDS gene is located at the Xq27/28 boundary [6], and contains nine exons spread over 24 kb [7,8]. It has been reported that 2.3 kb-IDS cDNA encodes a polypeptide of 550 amino acids showing high homology with the sulfatase protein family [8,9]. The synthesized IDS requires post-translational modification including removal of the signal sequence peptide, glycosylation, phosphorylation, proteolysis, and conversion of C84 to the catalytic formylglycine: the 76 kDa precursor is processed through intermediates to the 55 kDa and 45 kDa mature forms [10]. So far, at least 530 genetic mutations responsible for MPS II have been identified, and it is known that gross alterations lead to the severe form. However, missense mutations comprising about 30% of the MPS II mutations result in heterogeneous phenotypes ranging from the severe to the attenuated form.

As to human IDS, no crystal structure has been reported, and a few structural models have been constructed by means of homology modeling using crystal structure information of other sulfatases including arylsulfatase A and arylsulfatase B as templates [11–14]. In those studies, some missense mutations were found to be localized on the predicted IDS structure and the effects of amino acid substitutions were discussed. But the number of mutations discussed was very small.

In this study, we built a new structural model of human IDS by means of the homology modeling and molecular dynamics methods, and predicted structural changes in IDS caused by 131 amino acid substitutions responsible for MPS II, and examined the relationship between the mutant IDS structures and the respective clinical phenotypes.

## Methods

### Missense mutations in the *IDS* gene

In this study, we analyzed 131 missense mutations in the *IDS* gene for which the MPS II phenotypes have been clearly described (67 severe and 64 attenuated). These missense mutations, the respective phenotypes, and the references are summarized in **Table 1**, and other missense mutations in the *IDS* gene that were excluded in this analysis are summarized in **S1 Table**.

**Table 1:** IDS mutations, phenotypes, references, and structural characteristics for mutant IDS proteins.

Mutation	Phenotype^*^	Numbers of affected atoms			RMSD	ASA	Reference
		Main chain	Side chain	Active Site	(Å)	(Å^2^)	
p.D45N	Attenuated	30	40	13	0.04	1	Vafiadaki (1998) Arch Dis Child 79:237
p.R48P	Attenuated	81	107	0	0.086	30.6	Sukegawa (1995) Hum Mutat 6:136
p.Y54D	Severe	24	30	0	0.039	126.8	Karsten (1998) Hum Genet 103:732
p.S61P	Attenuated	80	67	0	0.071	2	Sohn (2012) Clin Genet 81:185
p.N63D	Attenuated	4	15	0	0.022	53.5	Karsten (1998) Hum Genet 103:732
p.N63K	Severe	28	43	0	0.038	53.5	Chang (2005) Hum Genet 116:160
p.L67P	Severe	24	24	0	0.045	3.2	Zhang (2011) PLoS One 6:e22951
p.A68E	Severe	188	205	0	0.125	2.1	Schroeder (1994) Hum Mutat 4:128
p.S71R	Severe	105	109	0	0.085	0.2	Li (1999) J Med Genet 36:21
p.S71N	Attenuated	30	29	0	0.04	0.2	Froissart (1998) Clin Genet 53:362
p.L72P	Severe	64	83	0	0.07	1.2	Zhang (2011) PLoS One 6:e22951
p.A79E	Attenuated	153	167	22	0.097	0	Karsten (1998) Hum Genet 103:732
p.Q80K	Severe	203	199	7	0.103	5.2	Gucev (2011) Prilozi 32:187
p.P86R	Severe	281	325	9	0.171	0	Hopwood (1993) Hum Mutat 2:435
p.P86L	Severe	114	118	6	0.096	0	Vafiadaki (1998) Arch Dis Child 79:237
p.S87N	Attenuated	10	10	2	0.025	0.4	Popowska (1995) Hum Mutat 5:97
p.R88C	Severe	189	222	29	0.108	0.4	Karsten (1998) Hum Genet 103:732
p.R88G	Severe	159	212	23	0.097	0.4	Froissart (1998) Clin Genet 53:362
p.R88H	Severe	23	37	16	0.032	0.4	Karsten (1998) Hum Genet 103:732
p.R88L	Severe	27	39	5	0.04	0.4	Karsten (1998) Hum Genet 103:732
p.R88P	Severe	40	63	16	0.051	0.4	Villani (2000) Biochim Biophys Acta 1501: 71
p.L92P	Severe	14	9	0	0.028	5.4	Popowska (1995) Hum Mutat 5:97
p.G94D	Attenuated	111	107	0	0.098	0	Hopwood (1993) Hum Mutat 2:435
p.R95G	Attenuated	126	147	0	0.086	34	Goldenfum (1996) Hum Mutat 7:76
p.R95T	Attenuated	87	97	0	0.071	34	Moreira da Silva (2001) Clin Genet 60:316
p.P97R	Severe	514	542	25	0.229	3.6	Sohn (2012) Clin Genet 81:185
p.L102R	Attenuated	129	142	8	0.127	0	Karsten (1998) Hum Genet 103:732
p.Y108C	Attenuated	0	0	0	0.002	131	Rathmann (1996) Am J Hum Genet 59:1202
p.Y108S	Attenuated	0	0	0	0.002	131	Zhang (2011) PLoS One 6:e22951
p.W109R	Severe	76	98	0	0.054	110.3	Chistiakov (2014) J Genet Genomics 41:197
p.N115Y	Attenuated	56	45	0	0.093	25.3	Vafiadaki (1998) Arch Dis Child 79:237
p.S117Y	Severe	134	127	0	0.094	34.6	Kim (2003) Hum Mutat 21:449
p.Q121R	Severe	221	241	9	0.146	24.6	Froissart (1998) Clin Genet 53:362
p.E125V	Attenuated	6	2	0	0.015	127.2	Rathmann (1996) Am J Hum Genet 59:1202
p.T130N	Attenuated	0	0	0	0.006	56.9	Boyadjiev (2012) Mol Genet Metab 105:S22
p.T130I	Attenuated	0	0	0	0.002	56.9	Zhang (2011) PLoS One 6:e22951
p.S132W	Severe	328	340	8	0.192	44.8	Jonsson (1995) Am J Hum Genet 56:597
p.G134R	Severe	146	171	9	0.11	1.4	Rathmann (1996) Am J Hum Genet 59:1202
p.G134E	Attenuated	61	78	9	0.1	1.4	Amartino (2014) Mol Genet Metab Rep 1:401
p.K135R	Attenuated	54	41	0	0.047	13.3	Hopwood (1993) Hum Mutat 2:435
p.K135N	Attenuated	28	38	0	0.033	13.3	Popowska (1995) Hum Mutat 5:97
p.H138D	Attenuated	355	365	21	0.155	13	Froissart (1998) Clin Genet 53:362
p.H138R	Severe	52	37	0	0.049	13	Chang (2005) Hum Genet 116:160
p.G140R	Attenuated	244	296	15	0.18	3.5	Kato (2005) J Hum Genet 50:395
p.S142F	Severe	0	0	0	0.002	84.8	Chistiakov (2014) J Genet Genomics 41:197
p.S142Y	Severe	0	0	0	0.003	84.8	Amartino (2014) Mol Genet Metab Rep 1:401
p.S143F	Severe	0	3	0	0.003	77.3	Vallence (1999) hum mut mutation in brief#233 online
p.D148V	Severe	71	70	3	0.072	105.2	Keeratichamroen (2008) J Inherit Metab Dis 31S2:S303
p.P157S	Severe	1	0	0	0.007	62.6	Amartino (2014) Mol Genet Metab Rep 1:401
p.H159P	Severe	64	69	4	0.086	46.6	Karsten (1998) Hum Genet 103:732
p.C171R	Attenuated	15	17	0	0.029	23.4	Kato (2005) J Hum Genet 50:395
p.N181I	Attenuated	1	0	0	0.009	121.9	Moreira da Silva (2001) Clin Genet 60:316
p.L182P	Attenuated	45	43	0	0.048	60.6	Isogai (1998) J Inherit Metab Dis 21:60
p.C184F	Attenuated	267	281	0	0.137	25.8	Rathmann (1996) Am J Hum Genet 59:1202
p.L196S	Attenuated	3	21	0	0.019	5	Jonsson (1995) Am J Hum Genet 56:597
p.D198N	Severe	53	99	0	0.062	0.6	Amartino (2014) Mol Genet Metab Rep 1:401
p.D198G	Attenuated	225	250	9	0.111	0.6	Karsten (1998) Hum Genet 103:732
p.A205P	Attenuated	11	9	0	0.032	2	Goldenfum (1996) Hum Mutat 7:76
p.T214M	Severe	1	0	0	0.003	82.4	Amartino (2014) Mol Genet Metab Rep 1:401
p.L221P	Attenuated	37	46	0	0.059	6.4	Hopwood (1993) Hum Mutat 2:435
p.G224A	Severe	1	9	0	0.037	3.3	Keeratichamroen (2008) J Inherit Metab Dis 31S2:S303
p.G224E	Severe	40	56	4	0.065	3.3	Karsten (1998) Hum Genet 103:732
p.Y225D	Attenuated	98	124	3	0.081	3.4	Sukegawa (1993) 3rd Inter Sympo on MPS, Essen
p.K227M	Attenuated	5	19	0	0.018	3.6	Isogai (1998) J Inherit Metab Dis 21:60
p.K227Q	Severe	7	27	0	0.025	3.6	Hopwood (1993) Hum Mutat 2:435
p.P228L	Attenuated	161	197	18	0.116	0.4	Vafiadaki (1998) Arch Dis Child 79:237
p.P228A	Attenuated	0	0	0	0.004	0.4	Sohn (2012) Clin Genet 81:185
p.P228Q	Severe	198	223	12	0.111	0.4	Amartino (2014) Mol Genet Metab Rep 1:401
p.P228T	Severe	20	19	1	0.039	0.4	Gort (1998) J Inherit Metab Dis 21:655
p.H229Y	Severe	14	25	4	0.027	15	Jonsson (1995) Am J Hum Genet 56:597
p.P231L	Attenuated	9	15	0	0.019	26.9	Gort (1998) J Inherit Metab Dis 21:655
p.R233G	Attenuated	9	17	0	0.021	85	Chistiakov (2014) J Genet Genomics 41:197
p.K236N	Attenuated	19	26	0	0.027	73.9	Gucev (2011) Prilozi 32:187
p.P261A	Attenuated	47	43	0	0.077	107.3	Sohn (2012) Clin Genet 81:185
p.N265I	Attenuated	41	57	0	0.044	0.5	Filocamo (2001) Hum Mutat 18:164
p.N265K	Severe	127	148	0	0.077	0.5	Chistiakov (2014) J Genet Genomics 41:197
p.P266R	Severe	105	140	0	0.086	6	Vafiadaki (1998) Arch Dis Child 79:237
p.W267C	Attenuated	54	54	0	0.057	82.9	Chang (2005) Hum Genet 116:160
p.Q293H	Attenuated	91	110	0	0.091	0.6	Schroeder (1994) Hum Mutat 4:128
p.K295I	Severe	7	16	0	0.015	71.2	Amartino (2014) Mol Genet Metab Rep 1:401
p.S299I	Attenuated	141	172	0	0.088	0	Kim (2003) Hum Mutat 21:449
p.S305P	Attenuated	63	67	0	0.054	30.7	Brusius-Facchin (2014) Mol Genet Metab 111:133
p.D308N	Attenuated	15	31	0	0.03	9	Isogai (1998) J Inherit Metab Dis 21:60
p.D308E	Attenuated	43	42	0	0.054	9	Gort (1998) J Inherit Metab Dis 21:655
p.T309A	Severe	0	0	0	0.003	55.2	Gort (1998) J Inherit Metab Dis 21:655
p.G312D	Attenuated	23	25	0	0.034	5.5	Amartino (2014) Mol Genet Metab Rep 1:401
p.S333L	Severe	67	73	21	0.072	0	Vafiadaki (1998) Arch Dis Child 79:237
p.D334N	Attenuated	22	23	2	0.034	0.8	Froissart (1998) Clin Genet 53:362
p.D334G	Severe	52	52	14	0.07	0.8	Li (1996) J Inherit Metab Dis 19:93
p.H335R	Attenuated	42	49	20	0.049	0.6	Froissart (1998) Clin Genet 53:362
p.G336E	Severe	235	291	30	0.159	0	Froissart (1998) Clin Genet 53:362
p.G336V	Severe	97	137	23	0.11	0	Kato(2005) J Hum Genet 50:395
p.W337R	Attenuated	84	111	18	0.057	0.2	Sukegawa (1995) Hum Mutat 6:136
p.L339R	Severe	37	69	1	0.042	3.8	Froissart (1998) Clin Genet 53:362
p.L339P	Severe	33	52	0	0.059	3.8	Amartino (2014) Mol Genet Metab Rep 1:401
p.G340D	Attenuated	20	17	0	0.024	4.2	Karsten (1998) Hum Genet 103:732
p.E341K	Severe	348	458	23	0.16	21	Vallence (1999) hum mut mutation in brief#233 online
p.H342Y	Attenuated	27	31	0	0.035	5	Vallence (1999) hum mut mutation in brief#233 online
p.W345R	Severe	25	32	1	0.03	0.4	Amartino (2014) Mol Genet Metab Rep 1:401
p.W345K	Attenuated	22	29	1	0.032	0.4	Popowska (1995) Hum Mutat 5:97
p.A346D	Attenuated	100	115	15	0.083	0	Olsen (1996) Hum Genet 97:198
p.K347E	Severe	249	251	18	0.109	0.8	Chang (2005) Hum Genet 116:160
p.K347T	Severe	90	97	16	0.072	0.8	Villani (1997) Hum Mutat 10:71
p.S349I	Severe	70	95	0	0.072	0.2	Gort (1998) J Inherit Metab Dis 21:655
p.P358R	Severe	320	323	8	0.123	2	Jonsson (1995) Am J Hum Genet 56:597
p.L403R	Severe	375	423	35	0.13	5.5	Froissart (1998) Clin Genet 53:362
p.L410P	Attenuated	21	28	0	0.043	0	Ben Simon-Schiff (1994) Hum Mutat 4:263
p.C422Y	Attenuated	312	311	9	0.197	0	Gort (1998) J Inherit Metab Dis 21:655
p.C422G	Attenuated	6	14	0	0.017	0	Hopwood (1993) Hum Mutat 2:435
p.C432R	Attenuated	33	35	0	0.043	113.1	Lualdi (2006) Biochim Biophys Acta 1762:478
p.C432Y	Severe	0	0	0	0.002	113.1	Karsten (1998) Hum Genet 103:732
p.Q465P	Severe	85	114	3	0.09	13.8	Hartog (1999) Hum Mutat 14:87
p.P467L	Severe	92	109	0	0.101	0.6	Moreira da Silva (2001) Clin Genet 60:316
p.R468Q	Severe	366	421	17	0.161	0	Vafiadaki (1998) Arch Dis Child 79:237
p.R468G	Severe	61	100	0	0.063	0	Hopwood (1993) Hum Mutat 2:435
p.R468L	Severe	82	117	0	0.06	0	Sukegawa (1995) Hum Mutat 6:136
p.R468P	Severe	289	301	0	0.13	0	Charoenwattanasatien (2012) Biochem Genet 50:990
p.P469H	Attenuated	96	118	4	0.063	0.2	Jonsson (1995) Am J Hum Genet 56:597
p.D478G	Attenuated	27	32	0	0.042	54	Schroeder (1994) Hum Mutat 4:128
p.D478Y	Severe	20	35	0	0.029	54	Karsten (1998) Hum Genet 103:732
p.P480R	Severe	116	145	0	0.088	42.9	Froissart (1998) Clin Genet 53:362
p.P480Q	Attenuated	39	47	0	0.07	42.9	Froissart (1998) Clin Genet 53:362
p.P480L	Attenuated	10	16	0	0.041	42.9	Froissart (1998) Clin Genet 53:362
p.I485R	Severe	170	236	0	0.141	155.6	Vafiadaki (1998) Arch Dis Child 79:237
p.I485K	Severe	1	0	0	0.005	155.6	Vafiadaki (1998) Arch Dis Child 79:237
p.G489D	Severe	62	68	0	0.064	1.8	Chang (2005) Hum Genet 116: 160
p.Y490S	Attenuated	6	7	0	0.024	74.7	Froissart (1998) Clin Genet 53:362
p.S491F	Severe	135	167	0	0.081	0.4	Sohn (2012) Clin Genet 81:185
p.W502C	Severe	23	20	0	0.03	2.2	Vafiadaki (1998) Arch Dis Child 79:237
p.L522P	Severe	55	65	0	0.063	0.8	Sohn (2012) Clin Genet 81:185
p.Y523C	Attenuated	12	17	0	0.023	8.4	Jonsson (1995) Am J Hum Genet 56:597

*Description of the phenotypes is according to the original papers.

### Structural modeling of the human wild type IDS protein

Because no IDS crystal structure has been reported, we built a model of human IDS using homology modeling server I-TASSER (zhanglab.ccmb.med.umich.edu/ I-TASSER/) [15]. To take protein structure fluctuations into consideration, we conducted molecular dynamics calculations using Gromacs (version 5.0.5) [16]. In the simulation, we used the AMBER99sb force field and SPC/E water models. At first, we performed structure optimization using the steepest-descent method. Then, we carried out equilibration in two phases (NVT and NPT ensemble). As the production run, we performed 10ns MD simulation using Parrinello-Rahman pressure coupling and V-rescale temperature simulation. In order to obtain a representative structure, we used the simulated annealing method.

### Structural modeling of mutant IDS proteins and calculation of the numbers of atoms influenced by amino acid substitutions responsible for MPS II

Structural models of mutant human IDS proteins responsible for MPS **II** were built by means of homology modeling using the wild type IDS protein model as a template. For that purpose, we used molecular modeling software TINKER [17], and energy minimization was performed, the root-mean-square graduate value being set at 0.05 kcal/mol・Å. Then, each mutant model was superimposed on the wild type IDS structure based on the Cα atoms by the least-square-mean fitting method. We defined that an atom was influenced by an amino acid substitution when the position of the atom in a mutant IDS protein differed from that in the wild type one by more than the cut-off distance (0.15 Å) based on the total root-mean-square distance (RMSD), as described previously [18]. We calculated the numbers of influenced atoms in the main chain (the protein backbone: alpha carbon atoms linked to the amino group, the carboxyl one, and hydrogen atoms of the molecule) and the side chain (variable components linked to the alpha carbons of the molecule), and in the active site (D45, D46, C84, K135, and D334). Then, average numbers of influenced atoms were calculated for the severe MPS II group and the attenuated one.

### Determination of the RMSD values of all atoms in the mutant IDS proteins

To determine the influence of each amino acid substitution on the total conformational change in IDS, the RMSD values of all atoms in the mutant IDS proteins were calculated according to the standard method [19]. Then, average RMSD values were calculated for the severe group and the attenuated one.

### Determination of the accessible surface area (ASA) of the amino acid residues of the IDS protein

The ASA of each amino acid residue in the structure of the wild type IDS was calculated using Stride [20] to determine the location of the residue in the IDS molecule. Then, the average ASA values of the residues for which substitutions had been identified in the severe group and the attenuated one were calculated.

### Statistical analysis

Statistical analysis to determine the differences in the numbers of influenced atoms, the RMSD, and the ASA values between the severe MPS II group and the attenuated one was performed by means of Welch’s *t*-test, and it was taken that there was a significant difference if p< 0.05.

### Coloring of the atoms influenced by different amino acid substitutions at the same residue on IDS

Coloring of the influenced atoms in the three-dimensional structure of IDS was performed for amino acid substitutions including P480R (phenotype: severe), P480L (phenotype: attenuated), D334G (phenotype: severe), and D334N (phenotype: attenuated) as representatives, based on the distance between the wild type and mutant to determine the influence of the amino acid substitutions geographically and semi-quantitatively according to the method previously described [18].

### Construction of a database including genotypes, clinical phenotypes, references and mutant IDS structures in MPS II

In order to help researchers and clinicians who study MPS II, we built a database including the genotypes, clinical phenotypes, references, and structures of mutant IDSs, according to the method described previously [21].

## Results

### Structural model of the human IDS protein and locations of the amino acid residues associated with MPS II

**Fig 1:**
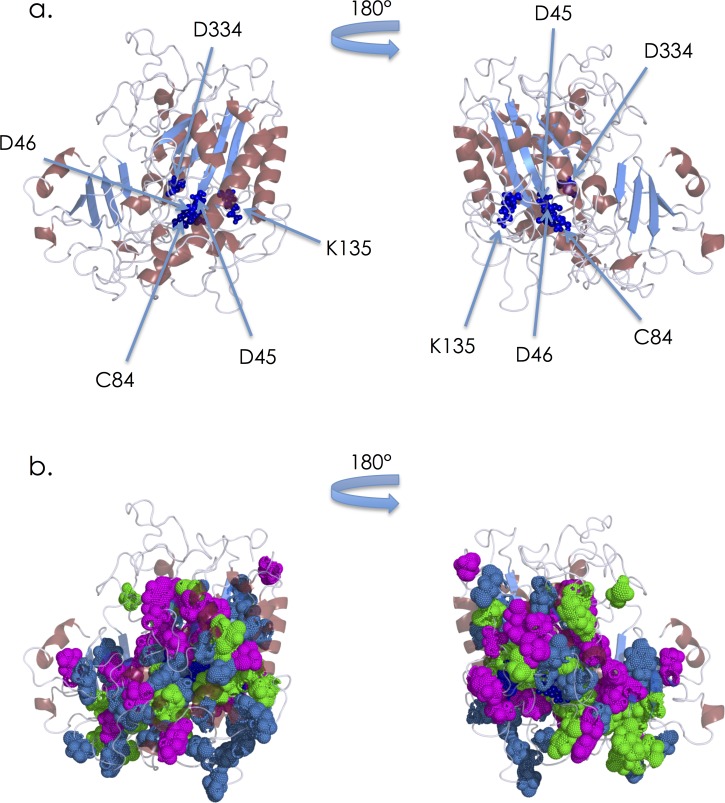
Predicted structure of human IDS **(a),** and positions of amino acid residues where substitutions responsible for MPS II have been identified **(b)**. The backbone is displayed as a ribbon model. **(a)** IDS is colored according to the secondary structure. Helices are colored red, sheets blue, and loops gray. The residues comprising the putative active site (D45, D46, C84, K135, and D334) are presented as small dark blue spheres. **(b)** The residues involved in substitutions responsible for MPS II are presented as spheres (severe: magenta, attenuated: blue, and both: green).

A homology modeling server was used to construct a structural model of the wild type IDS protein. For this calculation, several known structures were used as templates (pdb id: 2w8s, 2vqr, 3b5q, and 2quz). The results of the homology modeling revealed that IDS consists of alpha/beta folds, and that it contains two antiparallel beta-sheets (each sheet has 4 beta strands) with alpha helixes around them. The active site is located in the loop region near the N-terminal antiparallel beta-strand, and the D45, D46, C84, K135 and D334 residues comprising the putative active site [14] are indicated (**Fig 1A**). Then, to elucidate the relationship between the locations of the amino acid substitutions associated with MPS II and the respective phenotypes, we identified the positions of the residues in the wild type IDS protein structure (**Fig 1B**). The locations of the amino acid residues involved in the substitutions were widely spread over the enzyme molecule.

### Numbers of atoms influenced by amino acid substitutions associated with MPS II

**Fig 2:**
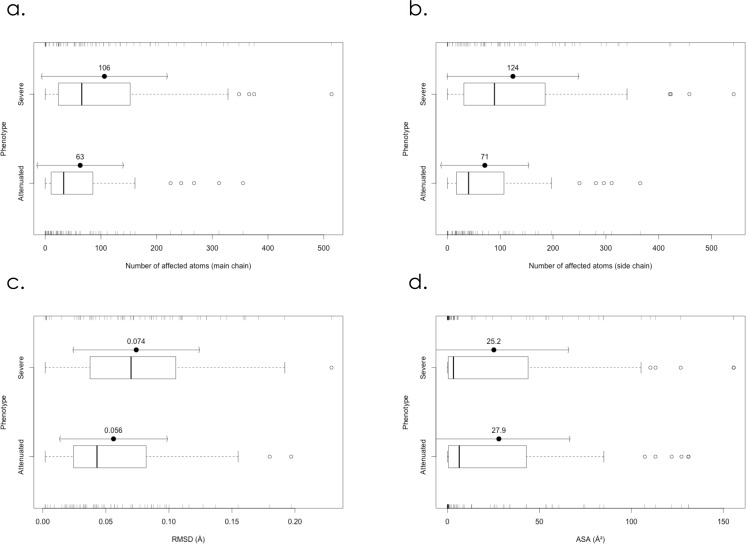
Boxplots of numbers of atoms affected by amino acid substitutions in the main chain **(a)** and the side chain **(b)**, RMSD values between mutant and wild type IDS proteins **(c)**, and ASA of amino acid residues where substitutions responsible for MPS II occur **(d)**. *Severe*: Severe group. *Attenuated*: Attenuated group.

Then, we constructed structural models of the mutant IDS proteins and calculated the number of atoms influenced by the amino acid substitution for each mutant model. The results are summarized in **Table 1**. In the severe phenotypic group, the average values (± standard deviation, SD) for the influenced atoms in the main chain and the side chain regarding the amino acid substitutions were 106 (± 113) and 124 (± 125), respectively. In particular, 42 of the 67 severe cases (63%) had 50 atoms or more influenced in the main chain. On the other hand, in the attenuated group, the averages (± SD) of the influenced atoms in the main chain and side chain were 63 (± 78) and 71 (± 84), respectively. Notably, regarding the main chain atoms, 39 of the 64 of the attenuated cases (61%) had 49 atoms or less affected. **Fig 2** shows the means ± SD and boxplots of the influenced atoms of the main chain (**Fig 2A**) and the side chain (**Fig 2B**) in the severe MPS II group and the attenuated one. Welch’s *t*-test revealed that there was a significant difference in the numbers of influenced atoms in both the main chain and the side chain (p<0.05) between the severe and attenuated groups. These results indicate that the structural changes caused by the amino acid substitutions responsible for severe MPS II were generally larger than those for attenuated MPS II. As to the influence of amino acid substitutions on the active site, the atoms of the residues comprising the putative active site were affected in 31 of the 67 severe cases (46%) and 17 of the 64 attenuated ones (27%).

### RMSD values between mutant IDS proteins and the wild type

To clarify the total structural change caused by each amino acid substitution, the RMSD value between the mutant IDS protein and the wild type was calculated, and the results are summarized in **Table 1**. The means ± SD and boxplots of RMSD values for the severe MPS II group and attenuated one are shown in **Fig 2C**. The average RMSD values (± SD) for the severe and attenuated groups were 0.074 (± 0.050) and 0.056 (± 0.043) Å, respectively. The Welch’s *t*-test showed that there was a significant difference in RMSD between the severe MPS II group and the attenuated one (p<0.05). These results indicate that structural changes in the severe MPS II group were larger than those in the attenuated one.

### ASA of the amino acid residues associated with MPS II mutations

To identify the locations of the residues associated with MPS II mutations in the IDS protein, the ASA values of the residues in the wild type structure were calculated and the results are summarized in **Table 1**. **Fig 2D** shows the means ± SD and boxplots of ASA for the severe group and the attenuated one. In the severe MPS II group, the average ASA value (± SD) was 25.2 (± 40.8) Å^2^. On the other hand, in the attenuated MPS II group, it was 27.9 (± 38.7) Å^2^. The results of the Welch’s *t*-test (p> 0.05) showed that there was no significant difference between the two groups.

### Effects of different substitutions at the same residue on the IDS structure and coloring of atoms affected in the molecule

**Fig 3:**
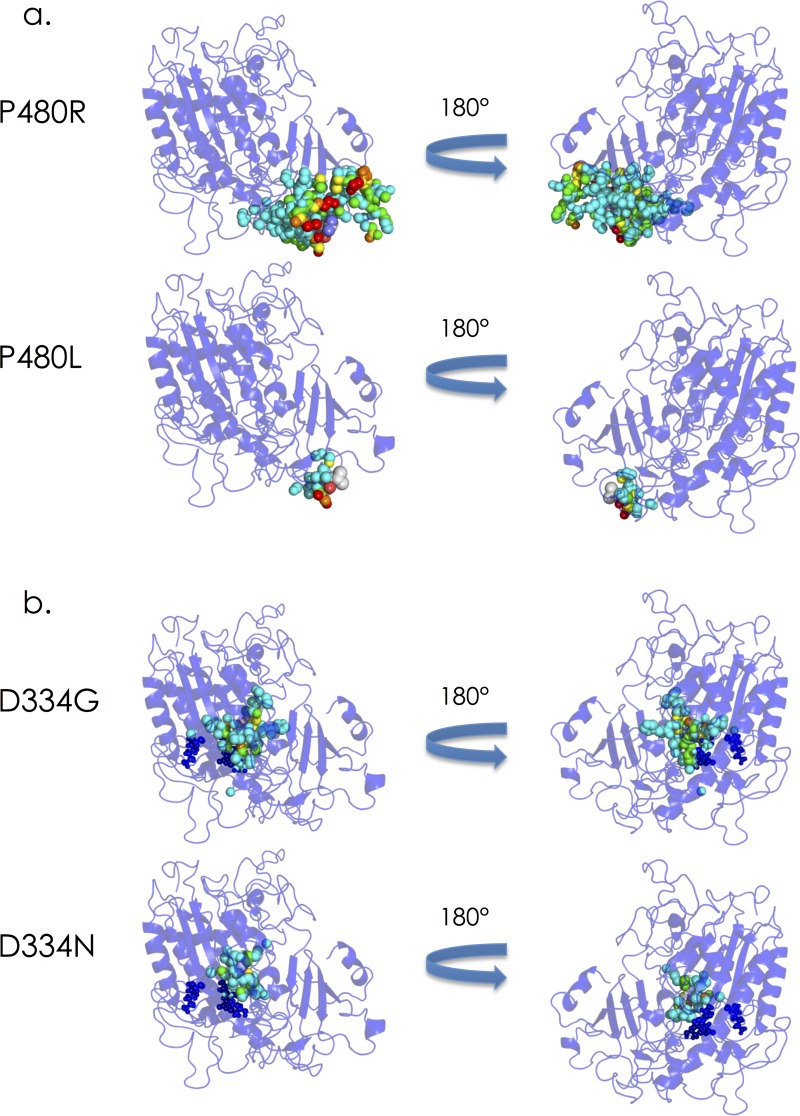
Coloring of the atoms influenced by amino acid substitutions, **(a)** P480R and P480L, and **(b)** D334G and D334N, in the three-dimensional structure of IDS. The backbone of IDS is shown as a blue ribbon model. The influenced atoms are presented as spheres. The colors of the influenced atoms show the distances between the wild type and mutant proteins as follows: 0.15Å ≤ cyan < 0.30 Å, 0.30 Å ≤ green < 0.45Å, 0.45 Å ≤ yellow < 0.60Å, 0.60 Å ≤ orange< 0.75Å, and 0.75Å ≤ red.

We next examined the effects of different substitutions at the same residue on the structural changes in the molecule and expression of the clinical phenotypes. There are at least 12 residues in the amino acid sequence of IDS at which different substitutions lead to different phenotypes (**Table 1**). For seven of those residues (N63, S71, G134, K227, N265, D334, and D480), the RMSD value and the number of affected atoms for the severe group were larger than those for the attenuated one. On the other hand, for 3 residues (H138, D198, and C432), the RMSD value and the number of affected atoms for the attenuated group were larger than those for the severe one. As to the other 2 residues (P228 and W345), it could not be determined for which phenotype the RMSD value and the number of affected atoms were larger than the other. Then, we examined the structural changes in IDS caused by P480R (phenotype: severe), P480L (phenotype: attenuated), D334G (phenotype: severe), and D334N (phenotype: attenuated) by means of coloring of the atoms affected. P480 is located near the molecular surface, and far from the active site. P480R is thought to cause a large structural change around the substituted residue, although it does not affect the active site, leading to the severe phenotype. The structural change caused by P480L is small and the influenced atoms are limited, leading to the attenuated phenotype (**Fig 3A**). On the other hand, D334 is a residue comprising the active site, and the D334G and D334N substitutions are both thought to directly affect the structure of the active site. The structural change in the former is larger than that in the latter, leading to the difference in phenotype (**Fig 3B**)

### Database of the genotypes, clinical phenotypes, references and mutant IDS structures in MPS II

**Fig 4:**
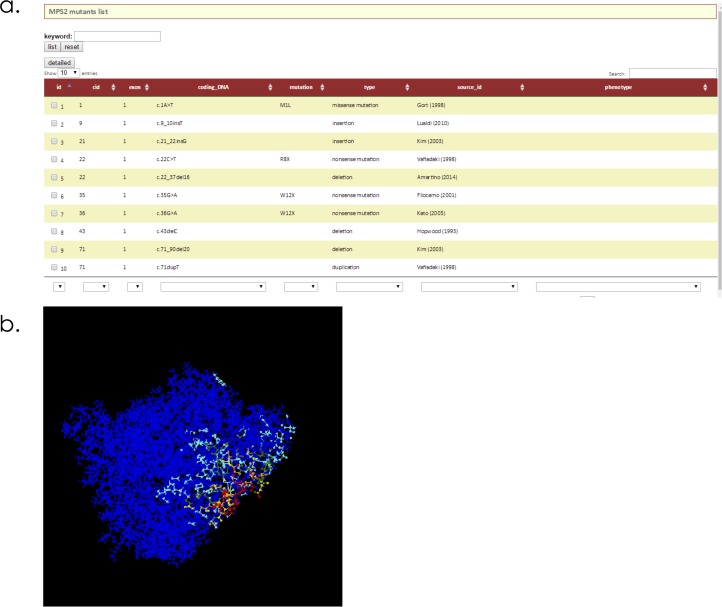
The list of MPS II gene mutations **(a),** and coloring of a mutant IDS protein with p.A68E **(b)** in the database (mps2-database.org).

We developed a database including the genotypes, clinical phenotypes, references and mutant IDS structures responsible for MPS II (mps2-database.org) (**Fig 4A**). The database provides readers with information on 530 MPS II mutations, which include 161 missense ones. The database is equipped with several tools. A text search tool is provided for searching the given text in selected fields of the database. Furthermore, using the control table option, users can search for MPS II mutations connected with specific phenotypes. A structural viewer is included in the database, which allows users to display the three-dimensional structures of the molecules using Jmol (**Fig 4B**). Furthermore, users can use many options for visualizing the structures of mutant IDS proteins.

## Discussion

Structural information on defective IDS proteins is important to elucidate the pathogenesis of MPS II, and is also useful for understanding the basis of the disease in each patient and for preparing a proper therapeutic plan for him or her.

So far, several structural models of the wild type IDS have been reported [11–14]. Unfortunately, they have not been registered in the Protein Data Bank (pdb), and thus we could not precisely compare our new model with them. However, as far as we examined the figures in their reports, it seems that there are no large differences in the whole structure between their models and ours, but there are small differences between them, i.e., in the loop regions of the molecule.

Regarding the locations of residues involved in amino acid substitutions, Kato et al. examined eight cases (phenotypes: 4 severe and 4 attenuated), and reported that the mutations found in the severe phenotype probably undergo direct interactions with the active site residues or break the hydrophobic core region of IDS, whereas residues of the missense mutations found in the attenuated phenotype were located in the peripheral region [13]. But our study, in which 131 missense mutations (phenotypes: 67 severe and 64 attenuated) were analyzed, revealed that there was essentially no difference in the localization of amino acid substitutions between the severe group and the attenuated one (**Figs 1B and 2D**).

In order to examine structural changes caused by the amino acid substitutions we calculated the numbers of atoms affected and the RMSD values. The results revealed that structural changes in the severe group were generally larger than those in the attenuated one (**Fig 2A, 2B and 2C).**

We examined structural changes caused by different substitutions at the same residue in the amino acid sequence of IDS and the phenotypes. Furthermore, structural changes caused by P480R, P480L, D334G, and D334N were examined as representative cases by coloring of the atoms affected in IDS. The results revealed that the structural changes influenced the disease progression.

A lot of expression studies have been performed, and the results revealed that the “attenuated” mutants expressed the precursor and small amounts of the mature IDS, resulting in residual enzyme activity, although the “severe” mutants expressed the precursor but no mature IDS, resulting in almost completely deficient enzyme activity [14,22–25]. Considering these findings, the majority of the “severe” missense mutants cause large structural changes and defects of the molecular folding, leading to rapid degradation and/or insufficient processing. On the other hand, the “attenuated” missense mutants generally cause small structural changes and moderate folding defects, leading to partial degradation of the enzyme protein and residual activity. Some mutants may directly affect the active site, leading to a decrease in enzyme activity according to the degree of the structural changes.

Finally, we built a database of IDS gene mutations responsible for MPS II, clinical phenotypes, references, and predicted structures of mutant IDSs. This database can be accessed via the Internet, being user-friendly.

In conclusion, we constructed structural models of the wild type and mutant IDS proteins by means of the latest homology modeling techniques, and showed the correlation between the structural changes and clinical phenotypes. This database based on the results of a structural study will help investigators and clinicians who study MPS II.

## Supporting Information

S1 Table Mutations which were excluded in the analysis. (XLSX)
